# Assessing Theory of Mind by Humor: The Humor Comprehension and Appreciation Test (ToM-HCAT)

**DOI:** 10.3389/fpsyg.2018.01470

**Published:** 2018-08-13

**Authors:** Simge Aykan, Erhan Nalçacı

**Affiliations:** Department of Physiology, Ankara University School of Medicine, Ankara, Turkey

**Keywords:** autistic traits, cartoons, humor, reliability, theory of mind, validity

## Abstract

Theory of Mind (ToM) may be defined as the ability to understand the mental states, such as beliefs, desires, intentions, and emotions, of others. Impairment of ToM ability leads to disorders with pathologies in social skills, such as autism spectrum disorder and schizophrenia. In addition to differences in ToM ability among patient populations, there is variation between neurotypical individuals. Unfortunately, ToM tasks are usually developed for children or patients with cognitive disorders and cannot detect variations in healthy adults. As an alternative tool, humor may be used. Humor plays a role in social communication and requires many different cognitive functions. Humor is believed to represent complex high-order cognitive processes. There are numerous types of humor; the most complex type is considered ToM humor, where an understanding of social/emotional content is necessary. Given the need for a ToM assessment test suitable for healthy adult populations, we developed a test for measuring humor comprehension and appreciation, with and without ToM content (ToM-HCAT). The present ToM-HCAT test is a performance test consisting of cartoons. The test measures perceived funniness, reaction time to perceived funniness decision, and meaning inference. Cartoons were selected after pilot studies involving 44 participants. Subscales were constituted according to expert views and confirmed by confirmatory factor analysis (*N* = 135). Goodness of fit values for the final 35-item test were acceptable to excellent: GFI = 0.97; AGFI = 0.97; NFI = 0.97; RFI = 0.97, and SRMR = 0.067. Both categories were internally consistent (α_1_ = 0.84, α_2_ = 0.94). External validity was assessed against autistic traits. One hundred and three participants completed the Autism Spectrum Quotient and were grouped by +0.5 standard deviations from the mean as high in autistic traits. The meaning-inference scores of the subscale with the ToM cartoons were significantly lower (*p* = 0.034) for the high autistic traits group, providing evidence of external validity. In conclusion, we developed and validated a test for assessment of ToM by humor comprehension and appreciation. We believe that the present test will be useful for the detection of variations in ToM ability in the healthy adult population.

## Introduction

As highly social beings, humans encounter a variety of interactions during their daily lives. Being successful in this environment requires insight into the social and emotional context and understanding of others’ intentions and aims, which is enabled by empathy. Empathy can be described as “*Any process that emerges from the fact that observers understand others’ states by activating personal, neural and mental representations of that state, including the capacity to be affected by and share the emotional state of another; assess the reasons for the other’s state; and identify with the other, adopting his or her perspective*” (de Waal and Preston, 2017, p. 498). In other words, empathy is characterized by the sharing of emotions and consideration of the perspectives of other people. Empathy may be divided into two categories: affective and cognitive (Singer, 2006; Zaki et al., 2012). Affective empathy involves the ability to match others’ emotions, while cognitive empathy refers to the ability to imagine how others feel. A type of cognitive empathy is theory of mind (ToM), which may be defined as the ability to understand the mental states of others, such as their beliefs, desires, intentions, and emotions (Wellman and Estes, 1986). In brief, ToM refers to the ability to understand one’s own, and others’, minds (Baron-Cohen, 2000). The importance of ToM may be illustrated by disorders in which ToM is impaired, such as autism spectrum disorders (ASDs) and schizophrenia (Baron-Cohen, 2000; Bora and Pantelis, 2013; Chung et al., 2014). Regarding autism, one of two main areas of impairment is that of social skills/communication: it is known that ASD patients exhibit the ability to share the emotions of others, but cannot mentalize (Smith, 2006), which is an indicator of impaired cognitive empathy. As underlying causes of disorders, ToM impairments have been studied extensively (Baron-Cohen, 2000; Losh et al., 2012; Chung et al., 2014; Sommer et al., 2018). Regarding schizophrenia, ToM impairments have been shown in unaffected relatives, ultra high-risk individuals, and first-episode patients as evidence of the trait-based nature of the disease (Bora and Pantelis, 2013; Lavoie et al., 2013).

In addition to differences in patient populations, variation in ToM abilities is observed among neurotypical individuals (Baron-Cohen et al., 2001a). However, ToM assessments are usually developed for children or cognitively disabled people (for a review, see Turner and Felisberti, 2017). As a result, the task used may not be sufficiently difficult for healthy adults with strong cognitive and social skills. These tasks have ceiling effects (near 100% accuracy) for healthy control participants (Corcoran et al., 1995; Gallagher et al., 2000; Brüne, 2003; Marjoram et al., 2005), which makes the detection of variations impossible. This limits the investigation of ToM in healthy populations, which is unfortunate as such investigations may shed light on its underlying mechanisms. Although the investigation of a cognitive mechanism in disabled populations provides useful insights, the results may be confounded by the presence of comorbid conditions. In addition, the disabled cognitive mechanism might be compensated by other processes, which will again lead to misinterpretation. Thus, investigation of the mechanism underlying a cognitive process should be accompanied by research in the healthy population. ToM variations are known to exist in healthy individuals (Baron-Cohen et al., 2001a), examples of whom include healthy first-degree relatives of schizophrenia patients (Janssen et al., 2003; Anselmetti et al., 2009; Bora and Pantelis, 2013) and relatives of individuals with ASD (Baron-Cohen and Hammer, 1997; Losh and Piven, 2007; Gokcen et al., 2009). Further, self-reported and neuroimaging data indicate variance in social cognition in the normal population (Hooker et al., 2010; Wagner et al., 2011; Regenbogen et al., 2015); however, behavioral data are lacking.

Current tasks used to measure ToM vary from social vignettes (e.g., false belief tasks, social animation tasks) to narrative fictional stories and films (e.g., strange stories tasks). There are limited number of ToM ability tests sensitive to variation in healthy population: the Reading the Mind in the Eyes Test (Baron-Cohen et al., 2001a), the Faux Pas Test (Stone et al., 1998; Gregory et al., 2002), the Yoni Test (Shamay-Tsoory and Aharon-Peretz, 2007), the DANVA (Nowicki and Duke, 2001), among others. As ToM is a highly complex process with cognitive and affective components that can be implicit and explicit, using a diversity of approaches for assessment is necessary. In addition to current tests, the use of humor represents a potential alternative method for ToM assessment in the healthy population. Humor might be described as anything that people say or do that is perceived as funny and makes others laugh (Martin, 2007). Humor is a way of communicating ideas, strengthening relations, improving group harmony, and expressing aggressiveness in a positive manner. Humor is the most flexible tool for social interaction. Therefore, it is important to express and understand humor to communicate more effectively. Humor is a stimulus encountered often in our daily lives, and the evaluation of humorous material may be considered similar to real-life situations, which makes it an appropriate tool for measuring ToM ability. The simplest form of humor is the pun, which uses visual or semantic resemblance, and the most complex form is ToM humor, which requires ToM abilities (Vrticka et al., 2013).

Humor processing consists of two stages: comprehension (the first stage) and appreciation (the second; Suls, 1972; Wyer and Collins, 1992; Vrticka et al., 2013). The most accepted theory of these is ‘incongruity detection and resolution,’ which states that humor requires the introduction of the incongruity as a violation of expectations, followed by a resolution associated with enjoyment (Shultz, 1972; Martin, 2007). Humor comprehension requires understanding of the context and detection of incongruity (Ruch, 1992; Coulson et al., 2006; Uekermann et al., 2007). Necessary cognitive processes for incongruity detection may vary from recognition of simple visual resemblance to mentalizing, which requires ToM ability. The second stage, humor appreciation, requires both integration of newly formed meaning in an amusing way and a positive emotional response (Wyer and Collins, 1992; Coulson et al., 2006; Uekermann et al., 2007). Therefore, humor appreciation represents a complex, high order process that involves cognitive, behavioral, physiological, emotional, and social components (Martin, 2007).

In addition to the previously mentioned ToM disability in ASD, another relevant trait is humor impairment. Asperger’s Syndrome (AS; one of the subtypes of ASD) was first defined by Hans Asperger in 1944. Individuals with AS are known to exhibit differences in terms of their perception of humor. This observation is supported by the fact that they have problems in understanding irony or sarcasm (Happé, 1995). Since Asperger’s work, researchers have verified humor-related deficits in ASD (Baron-Cohen, 1997; Emerich et al., 2003; Samson and Hegenloh, 2010). Samson and Hegenloh (2010) showed that humor appreciation in ASD individuals depends on the stimulus material. Appreciation was low for ToM cartoons, whereas no difference was observed for visual puns (Samson and Hegenloh, 2010). This result shows that humor appreciation is not reduced when ToM is not necessary.

Humor comprehension and appreciation differences in ASD would be expected to extend to the healthy population with autistic traits. Autistic traits are subthreshold deficits similar to those present in ASD, such as social interaction and communication deficits, as well as restrictive/repetitive behaviors (Constantino and Todd, 2003). The main difference between individuals with ASD and healthy people with autistic traits is the severity of the symptoms. A theory for ASD is that social adaptation and communication skills exhibit a normal distribution among the population, and individuals at the negative end cannot adapt to the social requirements of the population and, thus, constitute the ASD group (Constantino and Todd, 2003; Robinson et al., 2011; Lundström et al., 2012). Accordingly, it is widely known that ASD occurs as a spectrum in the diagnosed population; moreover, this spectrum is also observed among the general population (Baron-Cohen, 1995; Lundström et al., 2012; Ruzich et al., 2015). There is a genetic and biological overlap in the etiology of ASD and autistic traits (Bralten et al., 2018). Therefore, individuals at the end of this spectrum, with a high level of deficits, constitute the ASD group. Consistent with this view, studies examining autistic traits in the healthy population have been increasing in recent years. In addition, studies demonstrating differences in humor styles and appreciation (Eriksson, 2013; Rawlings, 2013) among healthy people with autistic traits similar to those in the ASD population have been reported.

To date, several instruments have been developed to assess the various dimensions of humor. These can be divided into two main groups: questionnaires and performance tests. As questionnaires are not relevant to the aim of this study, they are not discussed here (for a list of questionnaires, refer Ruch, 2007). Approximately 18 performance tests, with different measurement aims covering humor comprehension, appreciation, reasoning, and motivation, are have been constructed (for a list, see Ruch, 2007). None of these tests were developed directly for ToM assessment. However, studies assessing ToM using cartoons and jokes in patient populations and neurotypical individuals, which are not structured and have not been validated, have been reported (Happé et al., 1999; Gallagher et al., 2000; Samson et al., 2008). The stimuli are mostly unstructured cartoons or jokes that are used only in one study, reducing the possibility of replication.

Cartoons may be classified as one particular type of humorous material, i.e., static visual stimuli, and can be described as jokes in pictorial form (Nilsen and Nilsen, 2000). Cartoons may either consist of both text and pictures, or only pictures. The advantages of cartoons are that they do not depend merely on linguistic abilities, but also enable the depiction of characters’ emotions via their facial expressions or body postures. In contrast, in verbal humor, characters’ emotions must be described explicitly (Hempelmann and Samson, 2008). Henceforth, we will discuss studies using cartoons for ToM assessment, as our test consisted only of cartoons.

Cartoons with ToM content, which have been used in more than one study, were developed by Gallagher et al. (2000) and Marjoram et al. (2006). The stimuli consisted of cartoons in three categories; ToM, non-ToM, and jumbled pictures. The cartoons were grouped into categories by researchers and applied to 20 people before actual use. Meaning inference was assessed by open-ended questions and scored by a researcher as correct or incorrect. Another unstructured cartoon set was developed by Happé et al. (1999) and Snowden et al. (2003). Similarly to the above-mentioned study, the cartoons were divided into two categories (physical state and ToM) by researchers. Meaning inference for cartoons was assessed by open-ended questions and scored by researchers (Happé et al., 1999). One final example are the cartoons used by Samson et al. (2008). In this study, selected cartoons were pre-examined in several ways. Cartoons were categorized by five people in three categories as puns, those involving ToM, or as semantic, and cartoons with 90% total agreement were put into the related category. Twenty-one participants rated cartoons for funniness, complexity, and originality, with categories balanced regarding these parameters (Samson et al., 2008).

The research to date indicates that cartoon-based ToM assessment may be very useful; however, a structured, reliable, and validated test is currently not available. In addition, humor is a useful tool assessing ToM in healthy adults without a ceiling effect (Adolphs, 2003). Moreover, humor and ToM problems seem to co-occur, as seen in schizophrenia and ASD populations (Bozikas et al., 2007; Samson and Hegenloh, 2010). Hence, a test that measures both humor and ToM would be useful. Measuring both in the same test will provide an opportunity to understand whether these processes are disabled independently or in relation to each other. Finally, there is no structured humor test currently validated for use with a Turkish population.

Based on these demands, in the present study, we aimed to develop a humor test that measures humor comprehension and appreciation using cartoons with and without ToM content. More specifically, we aimed to create a task that: (i) was sensitive to differences in ToM ability in the healthy adult population, without a ceiling effect; (ii) was able to measure humor comprehension and appreciation ability with and without ToM ability; (iii) has adequate psychometric properties, being both reliable and valid; (iv) was objectively scored; and (v) was easy and quick to apply. Cartoons were presented, and time taken to decide whether the cartoon was funny or not (i.e., reaction time), scoring of funniness level (i.e., funniness score), answers for meaning of cartoons (i.e., meaning-inference score) were collected. The test was validated in relation to autistic traits.

## Methods and Results

### Participants

A total of 147 (79 females and 68 males, mean age = 22.56 years, *SD* = 4.41 years), undergraduate or graduate students from different faculties participated. As humor appreciation and comprehension change with aging (Greengross, 2013) we only included younger adults in order to constitute a more homogenous sample. The inclusion criterion was an age ranging between 18 and 35 years, and exclusion criteria were uncorrected visual impairment, a diagnosed neuropsychiatric disorder, and taking neuropsychiatric medication. The study was approved by the Ethical Committee of Ankara University School of Medicine.

### Test Development

The study was conducted in a series of four steps; for simplicity, the methods and results for each step are presented together. The first step of test development consisted of the selection of cartoons and piloting. The second step comprised experts grouping the cartoons. In subsequent steps three and four, reliability and validity were analyzed. In the test, three parameters for cartoons were assessed: reaction time (time taken to decide whether the cartoon was funny or not), funniness score (scoring of funniness level), and meaning-inference score (correct answers for meaning of cartoons). Confirmatory factor analysis was performed using AMOS 21.0 (Arbuckle, 2012), and all other analyses were performed using SPSS version 20.0 software (IBM Corp., 2011).

#### Step 1: Cartoon Selection

As either preference or dislike for sexual cartoons is known to correlate with personality characteristics (Ruch and Hehl, 1998) and was detected in all the factor analytic studies independent of the structural content (Eysenck, 1942; Herzog and Larwin, 1988; Ruch and Hehl, 1998), cartoons with high sexual content were excluded. Cartoons with low sexual, political, and violence content were collected from printed media or the internet. Colored cartoons were converted to black and white to exclude the facilitating effect of color on object recognition (Rossion and Pourtois, 2004), as this might cause a difference between colored and non-colored cartoons’ reaction times. Written cartoons with more than 70 characters were excluded as reading speed might have interfered with reaction times.

Participants were given instructions comprising a two-cartoon demo test, before the actual test session. Funniness scores and reaction times were collected using a computer. Funniness evaluation part of the test was presented in a dimly lit, soundproof room using a laptop with a 15.6^′′^, 1366 × 768 pixel resolution screen. MATLAB R2013a (MathWorks) with Psychtoolbox 3.0 (Kleiner et al., 2007) was used for presenting the cartoons and to record the reaction times and funniness scores. Participants were instructed as “A cartoon will appear on the screen and click to mouse when you decide if the cartoon is funny or not. Then a second screen will appear with numbers from one to seven, you should rate the funniness using a scale from one not funny to seven extremely funny.” Cartoons were projected on a gray background in randomized order. The time duration between cartoon presentation and mouse click was recorded as reaction time in seconds. The next screen display consisted of numbers from 1 to 7 and the words “Evaluate funniness level.” After the funniness level had been chosen, a new cartoon appeared on the screen.

**FIGURE 1:**
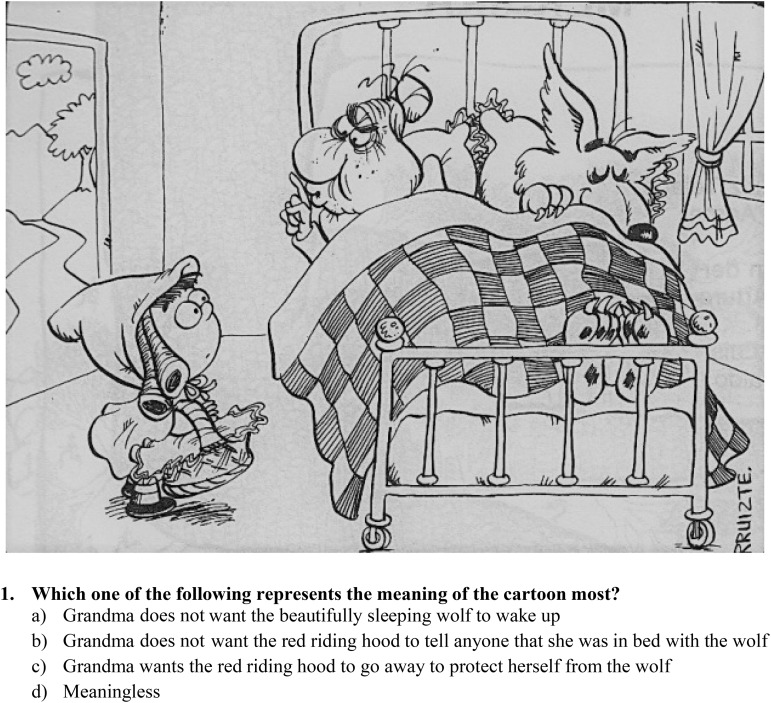
Sample question from meaning-inference test. Option b represents the main meaning. Options a and c are secondary meanings. Option d is “meaningless.” Cartoon is reprinted with permission from the Aydin Doğan Foundation. Copyright© 1986, Aydin Doğan Foundation.

The second part of the test is the meaning-inference test. It is a paper-based test and cartoons are presented in a booklet with one cartoon per page and with the question “Which one of the following represents the meaning of the cartoon most?” followed by four choices (see **Figure 1** for an example).

##### Step 1.1: Pilot study 1

A pilot sample of 12 individuals participated in this step (six females and six males, mean age = 23.75 years, *SD* = 3.33 years). Sixty cartoons were shown to the participants. The mean funniness-score was 3.07 ± 0.84 (range [1.50; 4.92]). Cartoons of funniness scores less than 2.5 (*n* = 22) were eliminated as they were considered to be unfunny for the target population. Mean reaction-time was 7.59 ± 2.30 s (range [3.61; 13.81]). Cartoons with a reaction time of greater than 12 s (*n* = 3) were eliminated due to their longer processing time, which may have indicated that they were more complex than the other cartoons. New cartoons were added to replace those removed. The next step then commenced with 60 cartoons.

##### Step 1.2: Pilot study 2

Thirty-two people participated in the second pilot study (16 females and 16 males, mean age = 26.63, *SD* = 5.11). Each participant scored the cartoons for funniness. In addition, each participant evaluated 20 cartoons for familiarity and meaning (10 evaluations per cartoon). They then answered two questions: the first question was “Have you seen this cartoon before?” and the second was “Write down the meaning of the cartoon in a single sentence. If you do not think it makes sense at all, you may write down ‘meaningless’.”

The maximum familiarity was 4/10, and cartoons with a familiarity of 2/10 or more (*n* = 20) were discarded. The study then continued with the remaining 40 cartoons. The mean funniness score and reaction time of each cartoon were calculated. Reaction times with *z*-scores over ± 3.0 were assigned as outliers as participants might have paused during the test or might have been distracted, and those values were excluded from the analysis. The mean reaction time was 7.03 s (*N* = 130–135, *SD* = 1.74, range [3.72; 10.73]). The mean funniness score was 3.57 (*N* = 135, *SD* = 0.46, range [2.65; 4.60]).

For the first phase of meaning-inference test development, answers to the above mentioned question were collected. In the second phase, four options were created by researchers based on these answers. Four options were designed as follows: one option was the main meaning, two options were secondary meanings, and one option was “meaningless.” The place of the main meaning was randomized in the first three choices and the fourth choice was always “meaningless.” The main meaning option was considered the correct answer. Every correct answer was scored as 1 point. The total number of points was referred to as the “meaning-inference score.” The meaning-inference test was presented to participants in two orders in opposite directions to eliminate the possible confounding effect of losing concentration toward the end. The test was applied to a pilot group of 10 participants. For three cartoons, the targeted choice was chosen by fewer than 50% of the participants and, thus, the options have been rearranged.

#### Step 2: Grouping of Cartoons Depending on ToM Content: The Experts’ View

To group cartoons into the two categories as ToM/Non-ToM, nine experts with at least a doctoral degree (social psychology *n* = 4, clinical psychology *n* = 2, developmental psychology *n* = 1, physiology *n* = 2) answered inquired to answer either yes or no to the question: “Do you think that social relations, values, feelings, and thoughts of people need to be understood in order to understand this cartoon?” The number of experts was chosen as an odd number, as this will always result in predominance of either the “yes” or “no” answer. Cartoons were assigned to the ToM category if the majority decision was “yes.” Cartoons with a majority of “no” were assigned to the Non-ToM (N-ToM) category. A Mann–Whitney *U* test indicated that the amount of “yes” votes that were associated with the ToM subscale (*Mdn* = 7) was significantly higher than the amount of “yes” votes that were associated with the N-ToM subscale (*Mdn* = 3), *U* = 0, *p* < 0.001. The ToM group consisted of 27 cartoons, whereas the N-ToM group consisted of 13 cartoons.

#### Step 3: Reliability

A group of 103 people participated in this part of the study (57 females and 46 males, mean age = 19.68, *SD* = 1.85). The funniness scores of 32 participants, who took part in pilot study 2 were included in the analysis. Therefore, data obtained from 135 participants (73 females and 62 males, mean age = 21.33, *SD* = 4.20 years) were used for reliability analysis.

The reliability of the subscales was assessed by three methods. First, Cronbach’s alpha coefficients were calculated as a measure of internal consistency (Cronbach and Meehl, 1955). For Cronbach’s alpha, values over 0.70 are accepted as good (Streiner and Norman, 1995; Kline, 2000). The Cronbach’s alpha coefficient was 0.84 for the N-ToM group and 0.94 for the ToM group, indicating good internal consistency. Both subscales showed good reliability.

Second, split-half reliability was used as another measure of internal reliability. A basic assumption of split-half reliability is that the two halves of the test should yield similar true scores and error variances (Brown, 1910; Spearman, 1910). In each subgroup, cartoons were divided into two groups (even- and odd-numbered). Spearman–Brown coefficients (*r_sb_*) were calculated. The coefficient for the N-ToM group was *r_sb_*= 0.83, and for the ToM group it was *r_sb_*= 0.95, indicating good consistency.

The third method involved the calculation of item-total correlations. Descriptive statistics, corrected item-total correlations of all items can be found in **Table 1**. The correlation coefficient is expected to be positive, above 0.30 (Nunnally and Bernstein, 1994). As shown in **Table 1**, item-total correlations were above 0.30 and positive, demonstrating the consistency of each item.

**Table 1:** Psychometric characteristics of the scale.

Item	Subscale	*M*	*SD*	CITC	SRC	SMC
1	N-ToM	3.26	1.89	0.54	0.58	0.34
2	N-ToM	2.91	1.80	0.58	0.64	0.42
3	ToM	2.96	1.72	0.62	0.67	0.44
4	ToM	3.36	1.70	0.62	0.66	0.43
5	ToM	2.72	1.53	0.56	0.60	0.36
6	N-ToM	3.38	1.83	0.59	0.62	0.39
7	ToM	3.99	1.95	0.57	0.59	0.34
8	ToM	3.43	1.95	0.51	0.54	0.29
9	ToM	3.75	1.87	0.68	0.68	0.46
10	ToM	3.69	1.66	0.61	0.61	0.38
11	N-ToM	3.12	1.84	0.48	0.48	0.23
12	ToM	3.34	1.77	0.69	0.71	0.50
13	N-ToM	4.12	1.81	0.45	0.47	0.22
14	ToM	3.50	1.89	0.64	0.66	0.43
15	ToM	3.57	1.82	0.64	0.62	0.39
16	ToM	3.60	1.90	0.62	0.62	0.39
17	ToM	3.86	1.80	0.62	0.62	0.39
18	ToM	3.69	1.94	0.60	0.62	0.39
19	ToM	4.04	1.82	0.70	0.71	0.50
20	N-ToM	3.40	1.88	0.55	0.60	0.36
21	ToM	4.58	1.85	0.52	0.57	0.32
22	ToM	3.56	1.76	0.51	0.54	0.29
23	N-ToM	2.66	1.57	0.48	0.50	0.25
24	ToM	3.81	1.68	0.61	0.62	0.39
25	ToM	3.80	1.90	0.58	0.60	0.35
26	ToM	3.72	1.91	0.59	0.61	0.37
27	N-ToM	3.60	2.00	0.36	0.48	0.23
28	ToM	3.59	1.76	0.48	0.50	0.25
29	ToM	4.57	1.74	0.63	0.64	0.41
30	N-ToM	2.93	1.69	0.58	0.58	0.33
31	N-ToM	4.00	1.77	0.50	0.52	0.28
32	N-ToM	3.28	1.88	0.46	0.51	0.26
33	ToM	4.17	1.97	0.62	0.62	0.39
34	N-ToM	4.04	1.90	0.54	0.60	0.37
35	ToM	3.95	1.89	0.49	0.52	0.27
36	ToM	3.83	1.87	0.64	0.67	0.45
37	ToM	3.42	1.98	0.62	0.64	0.41
38	ToM	2.78	1.68	0.57	0.55	0.31
39	ToM	3.10	1.75	0.57	0.59	0.35
40	N-ToM	3.94	1.81	0.62	0.69	0.48

*N = 134; M, mean; SD, standard deviation; CITC, corrected item-total correlation; SRC, standardized regression coefficient; SMC, squared multiple correlation; N-ToM, non-theory of mind; ToM, theory of mind*.

#### Step 4: Validity

##### Step 4.1: Construct validity

To evaluate the construct validity of the resulting model, confirmatory factor analysis was performed on the funniness scores. Data from 135 participants (the sample that was regarded within the reliability analysis) were used for confirmatory factor analysis.

Bartlett’s Sphericity Test and Keiser–Meyer–Olkin (KMO) were calculated as measures of the suitability of data for structure detection. For data to be considered suitable, the Bartlett’s test should be significant and the KMO value should be over 0.80 (Bartlett, 1954; Kaiser and Rice, 1974). The data were suitable for factoring as the Bartlett’s test was significant (*p* < 0.001) and the KMO value was 0.90.

According to the Mahalanobis distance measure, one participant was detected as a multivariate outlier and, thus, was excluded from the sample (Mahalanobis, 1936). Fit indices were estimated using the unweighted least-squares (ULS) method as kurtosis (163.23) and critical ratio (16.30) values suggested a non-normal distribution and data were ordinal in structure (de los Ángeles Morata-Ramírez and Holgado-Tello, 2013).

The assessment of model fit was based on several indices. The goodness-of-fit (GFI), adjusted goodness-of-fit (AGFI), standardized root mean square residual (SRMR), normed-fit index (NFI), and Bollen’s relative fit index (RFI) were used. The absolute fit indices (GFI and AGFI) calculate the proportion of variance that is accounted for by the model covariance. The SRMR shows the difference between the residuals of the sample covariance matrix and the hypothesized covariance model. The NFI shows the fit of the estimated model with the hypothesized model, and RFI considers inconsistency between the two models (Hooper et al., 2008). For GFI, AGFI, NFI, and RFI scores, >0.95 suggests a good fit whereas scores that are >0.80 suggest an acceptable fit (Bentler and Bonett, 1980; Bollen, 1989; Jöreskog and Sörbom, 1996). A SRMR <0.05 suggests a good data-model fit, while <0.08 suggests an acceptable fit (Hu and Bentler, 1999). Fit indices for the initial model are as follows: CFI = 0.97, AGFI = 0.97, NFI = 0.96, RFI = 0.96, SRMR = 0.070. The GFI, AGFI, NFI, and RFI suggested a good fit, and the SRMR suggested an acceptable fit.

Standardized regression coefficients of the variables were expected to be over 0.40, and all items were above that value. Squared multiple correlations should be over 0.30 but can be tolerated toward 0.10 if the other values are acceptable. Standardized regression coefficients and squared multiple correlations for the items are presented in **Table 1**. All regression values were above the expected value of 0.40. Squared multiple correlation values of 10 items were near but below 0.30. Items with low correlations were excluded from the model one by one, and fit indices were calculated. Items with lower indices were left out of the model. Three cartoons from the N-ToM group and two cartoons from the ToM group were excluded. The final model fit indices are as follows: CFI = 0.97, AGFI = 0.97, NFI = 0.97, RFI = 0.97, SRMR = 0.067. All the indices suggested an acceptable to good fit. The final test consisted of 35 cartoons: 10 cartoons from the N-ToM and 25 cartoons from the ToM.

Descriptives of test scores and reaction times for participants in this study group can be found in **Table 2**.

**Table 2:** Descriptives of test scores and reaction times for main study group.

Score	Subscale												
	N-ToM							ToM					
	Min	Max	*M*	*SD*	95% CI			Min	Max	*M*	*SD*	95% CI	
					LL	UL						LL	UL
Funniness	10	63	33.25	11.75	30.96	35.55		30	151	91.15	30.57	85.17	97.12
Reaction time^a^	2.11	12.67	7.08	2.11	6.66	7.49		2.49	12.92	7.21	1.74	6.87	7.55
Meaning-inference	2	10	6.67	2.00	6.28	7.06		5	24	17.68	3.53	16.99	18.37

*N = 135; N-ToM, non-theory of mind; ToM, theory of mind; Min, minimum; Max, maximum; M, mean; SD, standard deviation; CI, confidence interval; LL, lower limit; UL, upper limit; a, reaction time is in seconds*.

Descriptive statistics for the ToM-HCAT scores and reaction times, as well as comparisons of the subgroups, are given in **Table 3**. **Table 3** shows that no difference was found for funniness score, reaction time and meaning-inference score between cartoons in ToM and N-ToM subscales.

**Table 3:** Descriptives and comparison of ToM-HCAT scores and reaction times for cartoons in subscales.

Score	Subscale													
	N-ToM (*n* = 10)						ToM (*n* = 25)							
	Min	Max	*M*	*SD*	Median		Min	Max	*M*	*SD*	Median		*U*	*p*
Funniness	2.66	4.04	3.36	0.49	3.32		2.72	4.58	3.65	0.46	3.69		85.5	0.151
Reaction time^a^	4.37	9.81	6.96	1.97	6.85		3.72	10.13	7.08	1.68	7.49		120.0	0.872
Meaning-inference^b^	47	89	67.3	12.58	69.50		47	90	71.32	13.31	72.00		100.0	0.377

*N-ToM, non-theory of mind; ToM, theory of mind; Min, minimum; Max, maximum; *M*, mean; *SD*, standard deviation; CI, confidence interval; LL, lower limit; UL, upper limit; a, reaction time is in seconds; b, correct answer percentage of cartoons*.

##### Step 4.2: External validity

In the main study group, participants (*n* = 103) completed the Turkish version of the Autism Spectrum Quotient (AQ; Kose et al., 2010) for calculation of autistic trait scores in addition to the humor test. The maximum score for the AQ is 50 points; higher scores indicate higher levels of autistic traits (Baron-Cohen et al., 2001b). As the ASD group constitutes the higher end of the distribution for autistic traits (Robinson et al., 2011; Lundström et al., 2012), we adopted a similar approach in the present sample. Participants with AQ scores of +0.5 standard deviations were grouped as the high-autistic traits group (*n* = 37, mean AQ = 24.32, *SD* = 2.21, range [22; 28]). The rest of the population constituted the low-autistic traits group (*n* = 66, mean AQ = 16.58, *SD* = 3.14, range [6; 21]). A chi-square test was performed, and no difference was found for gender between high autistic traits (27:39 [m:f]) and low autistic traits (19:18 [m:f]) groups, χ^2^ (1, *N* = 103) = 1.05, *p* = 0.306. A Mann–Whitney *U* test indicated that age for the low-autistic traits group (*Mdn* = 19) was not significantly different from that for the high autistic traits group (*Mdn* = 20), *U* = 1119.5, *p* = 0.472.

The funniness-score and meaning-inference score on each of the two subscales were calculated as the sum of scores on that category. Scores were compared between groups and the means, 95% confidence intervals and comparisons of ToM-HCAT scores can be found at **Table 4**. **Table 4** shows that the meaning-inference score for the ToM category was lower for the high-autistic traits group (*Mdn* = 17) than for the low-autistic traits group (*Mdn* = 19); *U* = 914.5, *p* = 0.034. There was no difference for N-ToM and ToM funniness scores or N-ToM meaning-inference scores. The Spearman correlation between ToM meaning-inference scores and autistic traits scores was calculated and found to be low and non-significant, *r_s_*(102) = -0.14, *p* = 0.163. To test the robustness of this result the high-end split was further shifted to +1.0 SD. The high-autistic traits group (*n* = 19, mean AQ = 26.16, *SD* = 1.50, range [24; 28]) and the low-autistic traits group (*n* = 84, mean AQ = 17.82, *SD* = 3.68, range [6; 23]) were compared regarding the respective ToM meaning-inference scores. A Mann–Whitney *U* test indicated that the meaning-inference score for the ToM category was lower for the high-autistic traits group (*Mdn* = 17) than for the low-autistic traits group (*Mdn* = 19); *U* = 551.0, *p* = 0.035. Meaning-inference score of the ToM category was compared between females (*Mdn* = 18) and males (*Mdn* = 18) and there was no difference for gender *U* = 1180.0, *p* = 0.383.

**Table 4:** Descriptives and comparison of ToM-HCAT scores for high- and low-autistic traits groups.

Score	Low autistic traits (*n* = 66)						High autistic traits (*n* = 37)							
	Min	Max	*M*	*SD*	Median		Min	Max	*M*	*SD*	Median		*U*	*p*
**Funniness**														
N-ToM	10	52	33.67	11.54	34.5		15	63	32.51	12.25	30.0		1105.5	0.427
ToM	30	151	92.79	31.58	97.0		41	140	88.22	28.85	88.0		1106.0	0.429
**Meaning-inference**														
N-ToM	2	10	6.83	1.94	7.0		3	10	6.38	2.09	7.0		1068.5	0.289
ToM	5	23	18.08	3.68	19.0		11	24	16.97	3.17	17.0		914.5	**0.034**

*N-ToM, non-theory of mind; ToM, theory of mind; Min, minimum; Max, maximum; *M*, mean; *SD*, standard deviation; CI, confidence interval; LL, lower limit; UL, upper limit. Bold text indicates a statistically significant difference with a p-value less than 0.05*.

Reaction times for both categories were compared between groups. Descriptives and comparison of reaction times for high- and low-autistic traits groups can be found in **Table 5**. Results showed that the high-autistic traits group had longer reaction times for both subscales; however, no statistical difference existed between the low- and high-autistic traits groups. As reaction times might have been influenced by the number of characters in speech bubbles or by the amount of text in the cartoons, the relationships between text length and reaction time was analyzed. In the N-ToM subscale, 5/10 cartoons had speech bubbles or text. Similarly, in the ToM subscale, there were 25 cartoons, of which 10 featured speech bubbles. A Spearman correlation analysis between character count and reaction time showed a moderate positive correlation, *r_s_*(34) = 0.39, *p* = 0.022. Accordingly, the differences between character counts and reaction times for the N-ToM and ToM subscales were analyzed. A Mann–Whitney *U* test indicated that character count for the ToM subscale (*Mdn* = 0) was not significantly different from that for the N-ToM subscale (*Mdn* = 4.50), *U* = 120.5, *p* = 0.872. Similarly, the reaction time for the ToM subscale (*Mdn* = 7.49) was not significantly different from that for the N-ToM subscale (*Mdn* = 6.85, *U*= 120.0, *p* = 0.872).

**Table 5:** Descriptives and comparison of reaction times for high- and low-autistic traits groups.

Subcategory	Low autistic traits				High autistic traits				95% CI		
	*M*	*SD*	*n*		*M*	*SD*	*n*		LL	UL	*t*	*df*	*p*
N-ToM	6.78	2.00	66		7.60	2.24	37		-1.70	0.07	-1.85	67.64	0.069
ToM	6.97	1.68	66		7.64	1.80	37		-1.40	0.05	-1.87	70.15	0.066

*N-ToM, non-theory of mind; ToM, theory of mind; Min, minimum; Max, maximum; *M*, mean; *SD*, standard deviation; CI, confidence interval; LL, lower limit; UL, upper limit*.

## Discussion

In this study, we developed and validated a humor test, the ToM-HCAT, to assess humor appreciation and comprehension via the use of cartoons. This test comprises two different subscales: one subscale with ToM content and one subscale without ToM content. This theoretically assumed two-dimensional structure was analyzed by confirmatory factor analysis. The data showed an acceptable-to-good model fit, indicating good construct validity. Reliability measures were good and external validity was evident.

The ToM-HCAT is a performance test consisting of 35 cartoons, and has three outputs: (i) reaction time taken to decide whether the cartoon is funny or not; (ii) funniness score for each cartoon and subscale; and (iii) meaning-inference score for each cartoon and subscale. The reaction time reflects the processing speed of humor appreciation. The funniness score represents humor appreciation, and the meaning-inference score indicates humor comprehension. Within the ToM subscale the meaning-inference score reflects ToM ability by means of humor comprehension.

The test comprises cartoons with or without speech bubbles. In the first subscale, half (*n* = 5/10) of the cartoons have speech bubbles; in the second subscale, 10 out of 25 cartoons had speech bubbles. The distribution of cartoon types in groups is similar. For all cartoons, the text was limited to a maximum of 70 characters to exclude the effect of reading speed on reaction times. Further, this allows for reduced linguistic demands for comprehension. Although there was a moderate correlation between character counts and reaction time, no difference for character counts between subscales was observed, which makes it possible to compare these. All cartoons are black and white to exclude the confounding effect of color, especially on funniness scores and reaction times. The cartoons were chosen randomly from a large pool. The internet, printed cartoon books of Turkish cartoonists, and yearly books of the “Simavi International Cartoon Competition” (1983–1993) were used. Eighty-five cartoons were used in the study, and the final test consists of 35 cartoons; among these, 16 were published by international cartoonists, which also enables adaptation to other cultures.

Funniness decision consists of both humor comprehension and appreciation processes, although for appreciation it is not always necessary to comprehend (e.g., non-sense humor) (Ruch and Hehl, 1998). On the other hand, meaning-inference involves only the humor comprehension process. Accordingly, funniness and meaning-inference scores of ToM-HCAT should be considered as representing linked but different processes. In the meaning-inference test, participants choose the meaning from four options and it might happen that they comprehend the meaning after seeing the choices. In addition, it is possible that they may not have understood the main meaning of the cartoon in the previous funniness test. In our opinion, this does not decrease the importance of either result. This is because a funniness decision can be independent of comprehension; further, individuals may be unable to comprehend even after seeing the choices. Supporting this hypothesis, in the present study none of the participants achieved the maximum score for the meaning-inference test.

In addition to this, there are advantages to using a forced choice test. Current cartoon sets used in studies use subjective evaluations in which the researcher scores participants’ open-ended answers (Happé et al., 1999; Gallagher et al., 2000). The fact that the choices were selected by researchers in our study may be questioned; however, the choices were created after collecting explanations from a pilot group. Furthermore, there is no inter-rater reliability problems in the multiple-choice method. Inter-rater reliability refers to how similar the data collected by different raters are. If raters do not consistently agree in their scoring, then examiner specific factors may contribute unduly to observed score variability (Kline, 2011).

Another output of the test are reaction times for the funniness ratings, which provide the opportunity for evaluating the decision time. Decision time may be affected by cognitive processing speed, serving as a possible indicator of the efficiency of these processes. However, it should be noted that reaction time might have been influenced by numerous factors. For example, the complexity of the cartoons might have influenced the processing time. In the present study, we excluded cartoons for which reaction times were very long as such cartoons might have been overly complex. Another pitfall might have been that participants took a break or were distracted. To prevent this, we excluded reaction times with a very high *z*-score from the analysis. In our comparison group with autistic traits, reaction times were higher for the high-autistic traits group on both subscales; however, this difference was not significant. Similar results are presented in a cartoon Faux Pas Test. ASD participants took longer than neurotypicals to give their responses independent of cartoon types (Thiébaut et al., 2016). Longer reaction times might be related to the higher detail orientation of individuals with symptoms of autism (Dakin and Frith, 2005; Samson and Hegenloh, 2010). This finding is also an indicator that reaction times may be useful for measuring cognitive processing differences.

In the present study, we showed that individuals with higher autistic traits exhibit poorer humor comprehension if ToM is necessary for understanding the cartoon. It is widely known that the humor response of individuals with ASD differs from the response of neurotypical participants (Van Bourgondien and Mesibov, 1987; Baron-Cohen, 1997; Reddy et al., 2002; Samson and Hegenloh, 2010; Samson et al., 2013). This finding may be interpreted as a result of social communication deficits observed in this disorder (American Psychiatric Association, 2013). Regarding individuals with ASD, the response to humor varies according to the type of humor. Researchers have shown that ASD individuals do not appreciate humor created by socially inappropriate behavior (Reddy et al., 2002), and are unable to readily understand the other person’s humorous intention (Baron-Cohen, 1997). High-functioning autistic individuals may make jokes based on lexical or phonological contradictions; however, these tend to be under the age-appropriate level (Van Bourgondien and Mesibov, 1987). In support of our results, a study by Samson and Hegenloh (2010) showed that adults with ASD enjoy visual and semantic pun cartoons at similar levels as neurotypical individuals; however, these individuals exhibit difficulty in understanding ToM cartoons and provide less mentalistic explanations to humor consisting of ToM. In another study, it was shown that adolescents with high-functioning autism or AS performed worse than neurotypical individuals regarding the comprehension of cartoons and jokes (Emerich et al., 2003). We could not show a correlation between meaning-inference scores and AQ; however, this may have arisen from the relatively small sample size. Analysis using a higher number of participants may reveal a significant difference.

As the present population consisted of healthy people with autistic traits without a diagnosis of ASD, this study shows that impairment in ToM and humor extends to the healthy population with autistic traits. In support of this, differences in humor styles and appreciation have been reported among healthy individuals with autistic traits (Eriksson, 2013; Rawlings, 2013). For ToM impairment in healthy individuals, variation was shown by an implicit test: the Reading the Mind in the Eyes test. The test results were negatively correlated with autistic trait scores measured by the AQ (Baron-Cohen et al., 2001a). This result supports the present findings. We found that individuals with higher autistic traits score, as measured by AQ, exhibited poorer comprehension of cartoons with ToM, but not of cartoons without ToM.

To the best of our knowledge, there is no existing test that has the same structure and outputs as our humor test. The most similar test is the 3WD test of humor appreciation (Ruch, 1992): 3WD is another performance test that measures funniness and aversion to cartoons and jokes on a seven-point scale, with 35 items. Three categories of humor are present: non-sense, incongruity-resolution, and sexual. Although the tests are similar in the methods used to measure funniness, there are some differences between the ToM-HCAT and the 3WD test. The most important is that the present test aims to measure ToM processing, and cartoons have accordingly been categorized by their ToM content. To our knowledge, there is no other structured psychometric test that measures ToM ability by humor. A second difference is that we also measured humor comprehension in addition to appreciation.

As our aim was to develop a humor test that measures ToM ability, the finding of lower comprehension scores on ToM subscale cartoons for individuals with high autistic traits supports the validity of our test. Although a difference in funniness scores on the ToM subscale would be expected, we could not find any difference related to autistic traits. In contrast to the present results, a previous study mentioned above reported a significant difference in funniness among AS participants (Samson and Hegenloh, 2010). Although humor comprehension (resolution of incongruity) is considered a prerequisite for humor appreciation (Shultz, 1972; Suls, 1972), it has been suggested that only the detection of incongruity is necessary. This is supported by the appreciation of non-sense or slapstick humor, which does not involve incongruity resolution (Ruch and Hehl, 1998). Therefore, the lack of difference in funniness scores despite the low comprehension scores for the ToM category could be explained by this theory. It is proposed that individuals with high levels of autistic traits find incongruity sufficient for funniness, or that such individuals may perceive a different incongruity and/or resolution. Another difference with the current literature is that we could not show the gender difference in meaning-inference scores for ToM subscale. It is shown that women are superior compared to men in adult ToM tests (Baron-Cohen, 2002). However, in the study by Russell et al. (2007), men showed superior performance compared to women on both physical and mental state cartoons. The results emphasize the hypothesis that the differences in ToM tests could be task specific.

As our starting point was to develop a test to measure variability without a ceiling effect for ToM abilities in the adult healthy population, the findings suggest that our test can detect variability of ToM. Result cannot be attributed to humor ability, because the comprehension scores in the Non-ToM subscale did not show a difference in relation to high or low levels of autistic traits. None of the present participants achieved the perfect score of 25 out of 25 possible points on comprehension for the ToM subscale of the test; further, they scored almost the full range of possible scores of between 5 and 24 points, with a slightly left-skewed distribution. This variation suggests that the ToM-HCAT is sensitive to individual differences in ToM ability. This sensitivity in comparison with other tests may be attributable to the more real-world orientation of cartoons. Cartoons could be regarded as complex social scenarios that require social knowledge, and participants are required to make inferences about their meaning by both explicit mental state reasoning and spontaneous mental state inference. Furthermore, cartoons represent stimuli encountered in daily life.

### Limitations

There are several limitations to this study. First, the mean age of participants in the CFA analysis was 21.33 years, with the majority being between 18 and 22 years of age. Second, all participants were undergraduate/graduate students. The use of a more diverse sample is expected to enhance the validity of the current results. In particular, the age range should be wider. Another limitation is the application of the meaning-inference test on paper as a separate test. It may be beneficial to perform this test using a computer to ensure continuity of the entire test. Moreover, reaction times to meaning decisions should be collected, as these may be informative of processing time differences for ToM between individuals with high and low levels of autistic traits. Lastly, studies with larger numbers of participants are required. The present test was not validated using a diagnosed ASD population; this may appear to represent a limitation as this would be a gold standard for ToM disability. However, we validated the ToM-HCAT with autistic traits, which are more subtle than in individuals diagnosed formally with ASD. We showed that the test enables differentiation between these groups, thereby demonstrating its high sensitivity. Moreover, this test was developed to assess variations in ToM ability among the general population. This study, considering the small sample size, should be considered the first step of a scale development process. In future studies, a cross validation phase with a second and larger sample is necessary.

## Conclusion

In conclusion, a test for assessing ToM involving humor comprehension and appreciation was developed. The item and scale characteristics were good to excellent. The test was externally validated with autistic traits. It has multiple outputs and is suitable for use in future ToM assessment studies, especially in the healthy population, as it is sensitive to variations in ToM ability among neurotypical individuals. This test is expected to deepen our understanding of differences in ToM ability in the healthy adult population.

## Author Contributions

SA: design of the study, data collection and analysis, writing and contribution to all parts of the paper. EN: conception and design of the study, supervision of all parts of this project, contribution to all parts of the paper. Both authors contributed to the writing of the manuscript, read it critically, and gave consent to its publication.

## Conflict of Interest Statement

The authors declare that the research was conducted in the absence of any commercial or financial relationships that could be construed as a potential conflict of interest.
